# The Pathology of Cheyne-Stokes Breathing

**Published:** 1885-08-20

**Authors:** 


**The Pathology of Cheyne-Stokes Breathing**.—Some histological alterations in the bulb and pneumo-gastric nerves, to which changes he attributes the phenomena of Cheyne-Stokes respiration, have been recently discovered by Tizzoni (Lancet, January 31, 1885). This mode of respiratory rhythm consists in the presence of a prolonged pause followed by respiratory movements, at first slow and superficial, but gradually rising in frequency and increasing in depth, again slowly to decrease. So much misunderstanding has arisen on the subject of what is, and what is not, meant by this-named mode of respiratory rhythm that Tizzoni has done well to state precisely what it is that he meant. At the autopsy chronic inflammatory changes were found ascending the vagi, with blood, extravasation into the lymphatic spaces of the perineurium and endonerium. On the left side only the peripheral portion of the nerve was affected, while the right nerve was altered along the whole of its course, even to its origin. In the bulb the changes existed in the form of small foci, and were also more marked on the right side. The point where the alterations were most prominent was beneath the ependyma over the longitudinal furrow of the calamus scriptorius. In a second case, which was one of uræmia, the phenomena were observed for several days. The vagi were normal, but the superior half of the medulla oblongata presented lesions similar to those just described, though less pronounced and symmetrical.—*N. Y. Med. Rec.*

